# University and Leisure—A Commentary and My Broad Thoughts

**DOI:** 10.1007/s41978-022-00128-4

**Published:** 2023-01-17

**Authors:** Steve Olivier

**Affiliations:** Robert Gordon University, Aberdeen, Scotland

First, a few disclaimers– this Commentary is Written from a Personal Reflective Perspective. Although I draw on some Research, my Commentary is not research-based *per se*. Secondly, While I have Published in the Field of Leisure, my Research Interests have been both wide-ranging and Interdisciplinary, Embracing Physiology, Psychology, Ethics, and Morphology. Or, to Encapsulate it more Broadly, my Interests have Embraced the Potentially Linked Fields of Leisure studies/sports Science.

Another disclaimer is that I will, inevitably, use some examples from my own university. I have never been one of those vice-chancellors who, when invited to speak on something, spends the whole time discussing solely ‘their’ university (and you can tell from the quotation marks that I find the implication of ownership or dominion a bit distasteful). Having said all of that, examples are useful in an illustrative sense, and examples from personal experience can be proffered more authoritatively.

What I intend to do in this commentary is to present my thoughts on what leisure is, with some short remarks on the evolution of the academic discipline. From there I will comment on the relationship between leisure and work. I will explore what the role and obligations of universities (and their leaders) are towards staff, students and wider communities are (the order here is deliberate, as I shall explain). This will flow to a short reflection on work as leisure, and how we might retard what is perceived as growing managerialism and bureaucracy in universities. Specifically, I will ask how academic leaders might conceive of work as pleasure/leisure, referencing concepts such as the psychological construct of flow. This is also where I will introduce examples of interventions that we have put in place to enhance wellbeing, introduce flexibility, reduce bureaucracy, and harmonise working conditions. Finally, I will introduce some short reflections on the effects of the pandemic on leisure and wellbeing, and how we should embrace thoughts of equality and environmental sustainability into our conceptions of leisure and universities.

## Leisure, Work, and Universities

It is well to start this section by noting my adoption of a definition of leisure as that provided by Stebbins (2015, p.3), who holds that it is ‘uncoerced, contextually framed activity engaged in during free time, which people want *and choose* to do and, using their abilities and resources, actually do in either a satisfying or a fulfilling way’ (my italics). This explicitly recognises the notion of autonomy, and implicitly recognises the connection with work.

Marx (Veal, 2020) developed the notions of ‘realm of necessity’ and ‘realm of freedom’, and gaining access to the latter was a key motivator in seeking to overthrow capitalism. This is not the place to present historical developments in leisure studies, other than to say that several paradigms and typologies were proposed through the decades, particularly once working hours decreased after the second world war. Suffice to say, until the 1990s the notions of freedom and the relationship with work were pervasive features in leisure studies. Veal (2020) notes that in recent decades the downward trend in working hours in the USA was being reversed. Has working life and working time changed, at least in UK universities? Anecdotally, yes. A typical academic contract at UK universities is thirty-five hours per week, or seven hours per day. I suspect that it would be difficult to find a lecturer who agrees that this corresponds to the reality of working life.

Further, an oft-repeated complaint in university common rooms (where they still exist!) is that the pleasure derived from freedom and flexibility inherent in academic life has been replaced by longer work hours, managerialism, quality assurance, audit culture, and job insecurity. Fletcher, Carnicelli, Lawrence, and Snape (2016) cite several authors under an emergent theme of the instrumentalization of higher education, with learning replaced by ‘outcomes’, with academics becoming ‘producers’ and being redefined as dispensers of commodities. In support of this view, I recently received emails from two former colleagues saying ‘’I’m afraid that the technocrats, bureaucrats and trendy pedagogues are winning’, and ‘Work as pleasure seems like a very remote concept around here these days’. If this is the case, then it has implications for leisure, at least in the time available for it, and consequently for the good life as conceived of by Aristotle.

Affirming the above personally-expressed views, Harris (2012) holds that ‘The pleasures available in academic work can seem irrelevant in the in the emerging crisis’. If this is correct, what obligations do universities, and by implication university leaders have to provide a path to the good life for employees? Before considering how a re-conception of curriculum design and reviews of teaching, learning and assessment might help, and before providing examples of initiatives, I do want to stress that I believe we have an obligation to consider university employees as a whole. That is, we should attempt to destroy the binary divide that exists in many universities between ‘academic’ and ‘professional services staff’ – both should be treated equally in terms of access to leisure and pleasure, and this may involve harmonisation of working conditions and opportunities.

Focussing for the moment on academic work, traditionally this has been viewed as benefitting from significant autonomy, and of course this freedom is an integral part of leisure. Further than that though, Quinn (2007) talks of conceptual adventures and finding joy in the moment of intellectual creativity. A colleague of mine recently, in a chance corridor conversation, referred to such informal and unplanned encounters as ‘exquisite collisions’. All of these descriptions, and I see no reason why they need apply only to academic staff, imply something analogous to the concept of flow. This is described in sports psychology as an optimal stated underpinning peak performance (Partington, Partington and Olivier, 2009), where the experience itself is so enjoyable that people will do it… for the sheer sake of doing it (Csikszentmihalyi, 1975), and where the individual is free to act without the usual worries that plague our daily existence. Many would of course argue that this does not represent the predominant culture of higher education.

So can we move towards providing more pleasure associated with work, or even move towards a state approaching flow through a combination of re-engineering work and providing leisure opportunities? Without going into detail here (but see Robertson & Olivier 2020), I approach change leadership by first establishing both the purpose of change and, importantly, the principles that underpin it. So if, for example, might an underpinning principle of curriculum reform, or a review of teaching, learning and assessment practices be the provision of finding joy and fulfilment in work, supplemented by time and opportunities for leisure to contribute to the pleasure? I know what my answer would be, but I leave the question there for others to consider for their particular circumstances.

Many universities have comprehensive leisure, sport, and cultural offerings for staff and students. What follows here is a brief exposition of what we have done at the institution I currently lead. These are not exhaustive – rather they are a few examples, many of which I suspect are similar at other universities. Some of these initiatives are historic and well-established, while others were introduced during the Covid-19 pandemic. The titles are largely self-explanatory: free sports club, pool and gym membership for students (50% reduction for staff, rather than free, for tax reasons); lunchtime walk and talk sessions led by a trained walk leader; online app providing guidance for home exercise and wellbeing resources; pre-recorded and live online workouts; various intramural sports leagues and competitions; taster and beginner sessions for different sport activities; female only swimming sessions; additional rest days for staff; extra annual leave; Wellbeing Wednesday (no email or meetings from 1200 on Wednesdays; and so on. The next step was the introduction of a bureaucracy-free hybrid working policy, based on trust, where all staff members can work off-site for up to 50% of their formal work time. I say all staff, but there are a few categories of work that demand a full-time physical presence, and here we o compensate for lack of choice by, for example, increasing annual leave allowances.

## What Can Universities Do?

Increasing leave is one way to provide more freedom for leisure activities. The other ‘wellness’ initiatives mentioned earlier, while laudable and popular, do not however address the root cause of the erosion of leisure time, namely workload. This is where, I believe, university leaders need to focus their thoughts on how we approach job (re)design. A mindset of how we can design or redesign jobs of all types and all level to be fulfilling, and dare I say it, even pleasurable, should be an active consideration, alongside the more common approaches of productivity, outputs, and efficiencies, along with a performance recognition system that avoids over-working. Just as importantly, and in conclusion, I believe that we should actively foster a sense of empowerment and create a culture where individuals feel able to improve their own jobs, to be emboldened enough to come forward with suggestions that will improve their jobs so that they are as fulfilling as possible.
